# First-Trimester Plasmatic microRNAs Are Associated with Fasting Glucose Levels in Late Second Trimester of Pregnancy

**DOI:** 10.3390/biomedicines12061285

**Published:** 2024-06-10

**Authors:** Cécilia Légaré, Véronique Desgagné, Kathrine Thibeault, Frédérique White, Andrée-Anne Clément, Cédrik Poirier, Zhong-Cheng Luo, Michelle S. Scott, Pierre-Étienne Jacques, Patrice Perron, Renée Guérin, Marie-France Hivert, Luigi Bouchard

**Affiliations:** 1 RNA Institute, College of Arts and Sciences, University at Albany-SUNY, Albany, NY 12222, USA; cecilia.legare@usherbrooke.ca; 2 Département des Sciences de La Santé, Université du Québec à Chicoutimi, Saguenay, QC G7H 2B1, Canada; 3 Department of Biochemistry and Functional Genomics, Faculty of Medicine and Health Sciences (FMHS), Université de Sherbrooke, Sherbrooke, QC J1H 5N4, Canada; veronique.desgagne@usherbrooke.ca (V.D.); kathrine.thibeault@usherbrooke.ca (K.T.); andree-anne.clement@usherbrooke.ca (A.-A.C.); cedrik.poirier@usherbrooke.ca (C.P.); michelle.scott@usherbrooke.ca (M.S.S.); renee.guerin.csssc@ssss.gouv.qc.ca (R.G.); 4 Clinical Department of Laboratory Medicine, Centre Intégré Universitaire de Santé et de Services Sociaux (CIUSSS) du Saguenay–Lac-St-Jean, Hôpital Universitaire de Chicoutimi, Saguenay, QC G7H 5H6, Canada; 5 Département de Biologie, Faculté des Sciences, Université de Sherbrooke, Sherbrooke, QC J1K 2R1, Canada; frederique.white@usherbrooke.ca (F.W.); pierre-etienne.jacques@usherbrooke.ca (P.-É.J.); 6 Department of Obstetrics and Gynecology, Lunenfeld-Tanenbaum Research Institute, Mount Sinai Hospital, Faculty of Medicine, Institute of Health Policy, Management and Evaluation, University of Toronto, Toronto, ON M5S 1A1, Canada; zc.luo@utoronto.ca; 7 Centre de Recherche du Centre Hospitalier Universitaire de Sherbrooke (CR-CHUS), Sherbrooke, QC J1H 5N4, Canada; patrice.perron@usherbrooke.ca; 8 Department of Medicine, Faculty of Medicine and Health Sciences, Université de Sherbrooke, Sherbrooke, QC J1K 2R1, Canada; mhivert@partners.org; 9 Department of Population Medicine, Harvard Pilgrim Health Care Institute, Harvard Medical School, Boston, MA 02115, USA; 10 Diabetes Unit, Massachusetts General Hospital, Boston, MA 02114, USA

**Keywords:** microRNAs, fasting glucose, pregnancy, next-generation sequencing, plasma, ribo-hormone

## Abstract

Maternal blood glucose regulation adaptation to pregnancy aims to support fetal growth but may also lead to the development of gestational diabetes mellitus, the most common pregnancy complication. MiRNAs are small RNA molecules secreted and stable in the blood, where they could have paracrine hormone-like functions (ribo-hormone) and regulate metabolic processes including fetal growth and glucose metabolism. The objective of this study was to identify plasmatic microRNA (miRNAs) measured during the first trimester of pregnancy that were associated with glucose levels during a 75 g oral glucose tolerance test (OGTT) at ~26 weeks of pregnancy. miRNAs were quantified using next-generation sequencing in 444 pregnant women and replicated in an independent cohort of 106 pregnant women. MiRNAs associated with glucose levels were identified with the DESeq2 package. We identified 24 miRNAs associated with fasting glycemia, of which 18 were common to both cohorts (q-value < 0.1). However, no association was found between miRNAs and 1 h or 2 h post OGTT glycemia. To conclude, we identified 18 miRNAs early in pregnancy that were associated with fasting blood glucose measured 3 months later. Our findings offer new insights into the mechanisms involved in fasting glucose homeostasis regulation in pregnancy, which is critical to understanding how gestational diabetes develops.

## 1. Introduction

Gestational diabetes mellitus (GDM) is the most common pregnancy complication, affecting 6% to 20% of pregnancies worldwide [1]. GDM is diagnosed between the 24th and the 28th week of pregnancy by an oral glucose tolerance test (OGTT). Higher maternal glycemic levels, both while fasting and post-OGTT, across the whole spectrum of glycemia, have been associated with pregnancy complications [2] and adverse outcomes for both the mother and her offspring [3]. A better understanding of the early pregnancy mechanisms involved in maternal blood glucose physiologic regulation and those associated with the pathophysiology of GDM may inform the development of interventions to reduce its burden.

MicroRNAs (miRNAs) are short, single-stranded RNA molecules containing from 19 to 25 nucleotides that are secreted and stable in biofluids including plasma [4]. They target messenger RNA (mRNA), which leads to the decreased synthesis of the encoded protein [5]. MiRNAs are involved in many physiological processes, including pregnancy, and some miRNAs are expressed preferentially by the placenta [6]. These miRNAs are mainly in one of three clusters and are expressed by the placenta in a temporal manner throughout pregnancy: miRNAs from the chromosome 14 microRNA cluster (C14MC) are more abundant at the beginning of the pregnancy and then decline in the circulation progressively until delivery, while miRNAs from the chromosome 19 microRNA cluster (C19MC) and the miR-371-3 miRNA cluster become more abundant in the circulation as the pregnancy progresses [6]. These miRNAs, among others of placental origin, are released into maternal circulation, where they are carried by exosomes (and possibly other miRNA carriers, such as AGO2) [5]. This suggests that placental miRNAs secreted into maternal circulation could contribute to maternal physiological adaptation to pregnancy, including the decline in insulin sensitivity in the second half of pregnancy through feto–maternal intercellular communications [7]. This would confer a paracrine hormone-like function to circulating miRNAs (6). Indeed, in a previous study, we identified 18 miRNAs measured in the first trimester of pregnancy that can predict insulin sensitivity between the 24th and 28th weeks of pregnancy [8]. We also found miRNAs that are associated with maternal body mass index (BMI) during the first trimester of pregnancy that could be implicated in glucose regulation during pregnancy [9]. Additionally, we identified miRNAs that can predict women at risk of developing GDM [10].

Our objective was thus to identify plasmatic miRNAs that are detectable in the first trimester of pregnancy that can predict glucose responses to an OGTT performed between the 24th and the 28th weeks of pregnancy. We used glucose levels as a continuous trait as opposed to GDM diagnosis thresholds as this allowed for the association between miRNA and glucose regulation in pregnancy to be determined over the full range of glucose levels and because the impact of increased glucose levels on maternal and offspring health is linear.

## 2. Materials and Methods

### 2.1. Study Participants

For this study, participants were selected from two independent prospective birth cohorts. Details of the two cohorts were reported previously [8,10,11]. Briefly, the discovery cohort was composed of 444 women selected from the Genetics of Glucose regulation in Gestation and Growth (Gen3G) [12], and 112 women from the 3D cohort [13] were selected as the replication cohort in this study. The selection criteria were women of European descent with available plasma sample at first trimester of pregnancy and complete second trimester 75 g OGTT data for the 3D and Gen3G cohort, as well as at least one follow-up visit 3–5 years post-partum for the Gen3G cohort. The miRNA-sequencing data leveraged in this study were previously generated and used in prior publications [8,10,11] and are available on GEO (GSE216275 and GSE216997). The study was approved by the ethics review board of *CIUSSS de l’Estrie-CHUS* and all participants provided written informed consent.

BMI was calculated as weight (kg)/height (m^2^). The 75 g oral glucose tolerance test (OGTT) is a test in which 75 g of glucose is given to a patient in fasting state to measure how the body responds to a glucose load. Glycemia is measured before (fasting state), 1 h after, and 2 h after the glucose is ingested. Blood glucose excursion after ingestion (glucose area under the curve) is also a good marker of glucose tolerance or intolerance. The glucose hexokinase method (Roche Diagnostics, Indianapolis, USA) was utilized to quantify glucose levels in the Gen3G cohort, whereas glucose levels during the OGTT were collected from the medical records in 3D. In 3D, as in Gen3G, glucose was quantified in certified biochemistry laboratories that were part of the same health care system. Glucose levels were thus overall comparable between the two cohorts.

### 2.2. RNA Extraction and Library Preparation

In Gen3G and 3D, research staff collected plasma samples between the 4th and 16th week of pregnancy and stored samples at −80 °C upon RNA extraction. In brief, total RNA was extracted from 500 µL of plasma using the standard protocol of the MirVana PARIS kit (Thermo Fisher Scientific, Waltham, MA, USA, catalog # AM1556), followed by ammonium acetate precipitation, as described by Burgos et al. [14]. We applied the Truseq Small RNA Sample Prep kit (Illumina, BC, Canada; catalog # RS-200-0012) protocol, as adapted by Burgos et al. [14]. We randomized samples before both RNA extraction and library preparation. More details were reported by Légaré et al. [8,10,11].

### 2.3. Library Quality Control and Sequencing

Quality control of the libraries (assessment of the concentration, library length, and absence of primer dimers) was performed with either the Agilent High-Sensitivity DNA Kit (Agilent, Mississauga, ON, Canada; catalog # 5067-4626) on the Agilent 2100 Bioanalyzer or the Kapa Illumina GA with Revised Primers-SYBR Fast Universal kit (Kapa Biosystems, Wilmington, NC, USA; concentration) and the LabChip GX instrument (PerkinElmer, Waltham, MA, USA, catalog# CLS760672; library length and absence of primer dimers). Quantitative real-time PCR was utilized for library quantification.

Libraries from the Gen3G cohort were equimolarly pooled (HiSeq 2500: 7 pM final molarity; 12 libraries with different indexes per lane; HiSeq 4000: 10 pM final molarity; 20 libraries with different indexes per lane), denatured and clustered on single-read Illumina flowcells (catalog # GD-401-3001 and catalog GD-410-1001) using the standard protocol. Either an Illumina HiSeq 2500 or HiSeq 4000 sequencing platform with 50 cycles and 7 indexing read cycles were used for sequencing. Twelve samples were extracted twice and sequenced on the two platforms and their miRNA levels were highly correlated (Pearson correlation coefficient ≥ 0.94) [11], which confirms the reproducibility of the results on both platforms. For the 3D replication cohort, the Illumina NovaSeq platform was utilized. Each library pool (48 libraries per lane) was loaded at 225 pM on an Illumina NovaSeq 6000 S1 lane following the Xp protocol with 1 × 100 cycles (single-end mode).

### 2.4. Bioinformatics Analysis

The sequencing data were analyzed with the extra-cellular RNA processing toolkit (Excerpt) pipeline version 4.6 from Rozowsky et al. [15]. Outlier samples were identified via the visualization of raw read counts: 8 samples from the Gen3G cohort were excluded (7 with <500,000 and one with >25 M miRNAs reads), whereas 6 samples were excluded from the 3D cohort (<1 M miRNAs reads).

### 2.5. Statistical Analysis

The Mann–Whitney U test was used to compare participants’ characteristics between the two cohorts. MiRNAs associated with plasmatic glucose values (either fasting, 1 h post-OGTT, 2 h post-OGTT, or area under the curve (AUC) of the OGTT) were identified with the DESeq2 R package [16]. Default parameters were applied, including the Wald test and the collapseReplicates function that combines read counts from samples that were sequenced twice (n = 12). In model 1, the associations between miRNAs and plasmatic glucose levels were adjusted for the sequencing run and lane, as well as gestational age at the time of plasma collection. A second model also included maternal age and BMI at the time of plasma collection (Model 2). We corrected for multiple testing in Gen3G using the false discovery rate (FDR) method. Results were considered significant at an FDR q-value < 0.1 in Gen3G and were replicated in the 3D cohort when the direction of effect was the same in both cohorts and FDR q-values < 0.1 in 3D. Volcano plots were created with the EnhancedVolcano package [17].

### 2.6. KEGG Pathway Analysis

We conducted a Kyoto Encyclopedia of Genes and Genomes (KEGG) pathway analysis of the genes regulated by miRNAs associated with fasting glucose in both cohorts with mirPath v.3 software [18]. We used experimentally validated miRNA–mRNA interactions (Tarbase v7.0 database) [19]. We applied default settings of mirPath v.3, including a *p*-value threshold of 0.05, the application of an FDR correction, and the Fisher Exact Test (Hypergeometric Distribution) for enrichment analysis. We merged the results with the pathway union parameters.

## 3. Results

### 3.1. Participants’ Description

Participants from 3D were slightly older than those from Gen3G (31.3 ± 4.4, 31.3 vs. 28.5 ± 4.3 years old, respectively) and they were at a later gestational age at the time of plasma samples’ collection for miRNA analysis (11.9 ± 1.6 vs. 9.6 ± 2.3 weeks of pregnancy), as well as at the time of the 75 g OGTT (27.3 ± 1.4 vs. 26.4 ± 1.0 weeks of pregnancy). On average, the early pregnancy BMI of women included in both cohorts was in the overweight category (mean ≈ 26 kg/m^2^).

The characteristics of the participants are presented in Table 1.

### 3.2. Associations between miRNA Levels and Fasting Blood Glucose during OGTT

**Figure 1:**
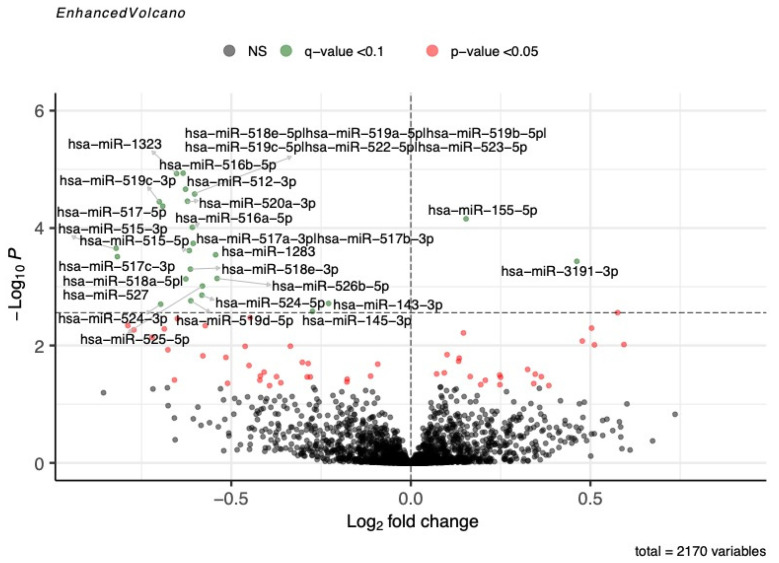
miRNAs associated with fasting glucose in Gen3G. Volcano plot showing first-trimester miRNAs nominally (*p*-values < 0.05; in red) or significantly (q-value < 0.1; in green) associated with fasting glucose levels measured at 24–28 weeks of pregnancy. miRNAs not associated with fasting glycemia are shown in grey. The q-value significance threshold (q = 0.1) is represented as a horizontal dotted line. Fold change represents a change in miRNA abundance per increase of one unit of fasting glucose in mmol/L. The model was adjusted for sequencing lane and run, as well as gestational age at the moment of plasma collection for miRNA measurement.

In Gen3G (discovery cohort), we detected 2170 miRNAs in maternal plasma collected in the first trimester of pregnancy. We first tested their associations with fasting glucose measured just before the 75 g OGTT performed between the 24th and the 28th weeks of pregnancy. We found a total of 24 miRNAs that were associated with fasting glucose (Model 1; q-value < 0.1; Figure 1 and Supplementary Table S1): two positively (Log_2_(Fold change) (L2FC) = 0.154 to 0.462) and 22 negatively (L2FC = −0.229 to −0.821).

Eighteen (75%) miRNAs were replicated in 3D (q-value < 0.1; Table 2): one positively (L2FC = 0.205) and seventeen negatively (L2FC = −0.390 to −1.197) associated with fasting glycemia. “Fold change” corresponds to changes in miRNA levels (read counts) for an increase of one mmol/L of glucose, which explains the relatively small L2FC we are reporting.

Fifteen of these miRNAs were located within the placental miRNAs cluster on chromosome 19 (C19MC, Table 2). All but miR-155-5p were negatively associated with fasting blood glucose levels and only miR-515-3p was detected in less than 80% of the 542 tested samples.

After considering maternal age and BMI in the analyses (Model 2), 10 miRNAs remained nominally associated with fasting blood glucose in both Gen3G and in 3D, with a reduced strength of association based on their fold change (Table 3). One participant had high fasting glycemia (7.3 mmol/L). We obtained similar results (sensibility analysis; Supplementary Table S2) after removing this participant from the analysis.

### 3.3. Associations between miRNA Levels and 1 h and 2 h Post-OGTT Glucose Levels

We then tested whether any of the miRNAs quantified in maternal plasma at the first trimester of pregnancy were associated with glucose levels measured 1 h and 2 h after 75 g glucose ingestion (75 g OGTT). Only miR-143-3p was associated with 1 h post-OGTT glucose levels in Gen3G (L2FC = −0.085, q-value = 0.0010; Supplementary Table S3). A total of 14 miRNAs were associated with 2 h post-OGTT glucose levels in Gen3G (q-value < 0.1; Supplementary Table S4): eight positively (L2FC = 0.036 to 0.210) and six negatively (L2FC = −0.051 to −0.425). After further adjusting the model for maternal age and BMI, 12 miRNAs remained associated with 2 h post-OGTT glycemia in Gen3G (q-value < 0.1; Supplementary Table S4). Two miRNAs were associated with the AUC of the OGTT glucose levels: miR-143-3p (L2FC = −0.066; q-value = 0.0003) and miR-484 (L2FC = 0.032; q-value = 0.0855; Supplementary Table S5). None of these miRNAs were replicated in 3D (Supplementary Tables S3–S5).

### 3.4. Pathway Analysis of miRNA Associated with Fasting Glucose

Finally, we ran a KEGG pathway analysis to understand how the miRNAs we identified could contribute to regulating fasting glucose levels. Interestingly, we observed that five of the eighteen miRNAs associated with fasting glucose were implicated in the extracellular matrix–receptor interaction pathway (*p* = 5.88 × 10^−9^); no other pathways were identified as being targeted by those miRNAs. More specifically, the targets of these five miRNAs, *COL5A2* (hsa-miR-516b-5p), *LAMB1* (hsa-miR-512-3p), and *COL6A2* (hsa-miR-516a-5p), were *COL1A1* (hsa-miR-518a-5p), as well as *COL1A2* and *FN1* (hsa-miR-145-3p) genes.

## 4. Discussion

In this study, we identified 18 miRNAs quantified between the 4th and 16th weeks of pregnancy in two independent cohorts that were associated with fasting blood glucose levels measured between the 24th and 28th weeks of pregnancy. However, we did not identify robust associations between first-trimester plasma miRNAs and 1 h post-OGTT, 2 h post-OGTT, and OGTT-AUC glucose levels. The presence of an association with fasting glycemia, and the absence of association with glucose levels measured after the OGTT suggests that circulating miRNAs are either markers of maternal BMI or they are part of the fasting regulation of glucose that is mainly dependent on hepatic glucose handling.

**Figure 2:**
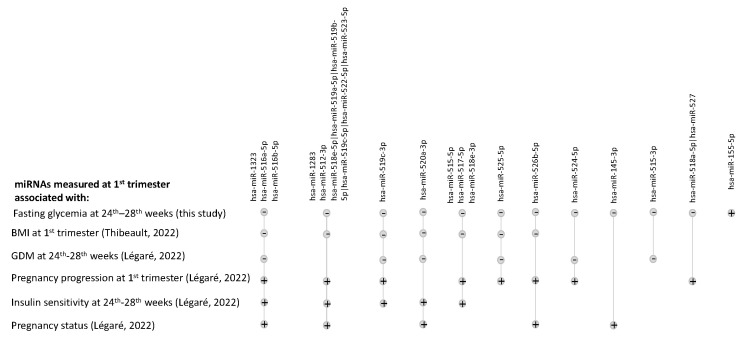
Upset plot listing fasting-glucose-associated miRNAs previously associated with other traits in prior studies from our group. miRNAs that were found to be associated with fasting glucose in this study are shown on the top line, and the intersections show associations with other traits/phenotypes during pregnancy in our prior studies of Gen3G. The direction of the association is represented by a plus sign for listed traits/phenotypes positively associated with miRNAs and a minus sign for those negatively associated with miRNAs [8,9,10,11].

Although some miRNAs we identified were relatively low in abundance based on normalized read counts, all were detected in most (>80%) of the 542 samples tested. In previous studies, 17 of the 18 miRNAs (94%) associated with fasting blood glucose in the current study were also associated either with maternal BMI in the first trimester of pregnancy [9], GDM [10], or insulin sensitivity (Matsuda Index) assessed between the 24th and 28th weeks of pregnancy [8], as well as with pregnancy status or progression during the first trimester [11]. Figure 2 shows with which of these phenotypes they were also associated.

It is noteworthy that all but two miRNAs (hsa-miR-155-5p and hsa-miR145-3p) are expressed from the placental C19MC. C19MC miRNAs are known to become more abundant as the pregnancy progresses [6]. Putative functions of the C19MC miRNAs include trophoblast differentiation and maternal–fetal communication [20]. Altogether, this supports the idea that miRNAs, and more specifically those secreted in maternal circulation by the placenta, may contribute to the physiologic maternal glucose metabolism adaptation to pregnancy and/or to the pathophysiology of GDM.

Interestingly, all miRNAs but hsa-miR-155-5p were negatively associated with fasting blood glucose levels, suggesting that those miRNAs could contribute to a decrease in blood glucose concentration by reducing glycogenolysis, hepatic gluconeogenesis, or insulin resistance. Hsa-miR-155-5p was the only miRNA positively associated with blood fasting glucose and was found to improve insulin sensitivity as well as promoting glucose uptake [21], which could be in line with our results if secreted in a counter-regulation feedback loop context. A decrease in its levels in the serum, peripheral blood mononuclear cells, or white blood cells has been associated with type 2 diabetes as well as GDM, but other studies also found it to be upregulated in the plasma of type 1 and 2 diabetes [21].

In our analyses, when maternal age and BMI were taken into account in the analyses, the effect sizes were substantially attenuated based on the observed fold changes, and none of the q-values < 0.1. Unravelling the role of maternal age and overweight and obesity (BMI) in the development of GDM is not straightforward, but our results suggest that maternal BMI is a confounder. These associations were lost after correction for maternal age and BMI, as with hsa-miR-1283, hsa-miR-518e-3p, hsa-miR-526b-5p, and hsa-miR-525-5p, which may be explained by their association with maternal BMI, as we have previously reported [9]. In these cases, their role in glucose metabolism regulation might be mediated by or linked to the maternal obesity status early in pregnancy, but BMI could also be a confounder and affect both miRNA and glucose metabolism separately.

Metabolic pathway analyses revealed that the miRNAs associated with fasting glucose regulate genes implicated in the extracellular matrix–receptor interaction pathway. This pathway is of potential interest in the glucose metabolism as the modulation of extracellular matrix (ECM) was associated with insulin resistance [22]. In brief, the deletion of integrin β1 or α2 reduced insulin resistance in two mice models [22]. Although none of our miRNAs are known to target integrins, their association with the ECM–receptor interaction pathway suggests a mechanism by which they could contribute to regulate glucose metabolism.

### Strengths and Limitations

To the best of our knowledge, this is the first study associating the miRNAs measured in early pregnancy and fasting glycemia assessed, on average, three months later. Using next-generation sequencing, we were able to robustly identify miRNAs associated with fasting glucose in two independent prospective birth cohorts. Our study also has limitations: all participants were of European descent; further studies are warranted to understand the generalization of the finding to other ethnic populations. Also, we did not validate, via functional analyses, the role of miRNAs in glucose regulation. Although next-generation sequencing is a robust and well-accepted method to identify and quantify miRNAs [23], other analytical strategies, such as real-time and digital PCR, may be applied to assess the plasma concentration (copies/µL) of these miRNAs in future studies. These strategies may be more applicable in a clinical setting, as they will be both cheaper and faster. The cost of measuring miRNAs should also be compared to that of performing a glucose challenge test during the first trimester of pregnancy. These miRNAs could be used to identify women at higher risk of maintaining higher fasting glucose levels in pregnancy. A closer follow-up of those women, as well as diet and exercise recommendations, could help women to maintain a better fasting glucose profile throughout pregnancy and prevent the development of GDM.

## 5. Conclusions

We identified miRNAs quantified during the first trimester that were associated with fasting blood glucose measured between the 24th and the 28th weeks of pregnancy. These miRNAs could contribute to glucose regulation during pregnancy, suggesting a new avenue in materno–fetal (placenta) communication as well as new targets that could be leveraged in the prevention of gestational diabetes and its consequences for both the mothers and their offspring.

## Figures and Tables

**Table 1:** Characteristics of study participants.

Characteristics	Gen3G (n = 436) Mean ± SD (Range)	3D (n = 106) Mean ± SD (Range)	*p*-Value a
1st trimester variables			
Gestational age (weeks)	9.6 ± 2.3 (4.1–16.3)	11.9 ± 1.6 (5.6–15.6)	<0.001
Age (years)	28.5 ± 4.3 (18.0–47.0)	31.3 ± 4.4 (22.0–45.0)	<0.001
Body mass index (kg/m^2^)	25.9 ± 6.0 (16.1–54.1)	26.4 ± 6.6 (18.1–47.2)	NS
1 h post-GCT glycemia (mmol/L) b	5.7 ± 1.4 (2.6–10.2)	NA	NA
2nd trimester variables			
GDM n (%)	13 (56)	45 (42)	NA
Gestational age (weeks)	26.4 ± 1.0 (24.1–29.4)	27.3 ± 1.4 (24.4–30.0)	<0.001
Fasting OGTT glycemia (mmol/L)	4.2 ± 0.4 (3.4–7.3)	4.7 ± 0.5 (3.7–6.6)	<0.001
1 h post-OGTT glycemia (mmol/L)	7.3 ± 1.7 (3.6–13.0)	8.9 ± 1.6 (4.7–12.3)	<0.001
2 h post-OGTT glycemia (mmol/L)	5.9 ± 1.4 (3.0–11.4)	7.1 ± 1.4 (3.8–11.5)	<0.001

a = Mann–Whitney U test; b = data available only for 403 Gen3G participants. Abbreviations: GCT: 50 g glucose challenge test; GDM: gestational diabetes mellitus; NA: not applicable; NS: not significant; OGTT: 75 g oral glucose tolerance test; SD: standard deviation.

**Table 2:** miRNAs associated with fasting blood glucose in both cohorts.

miRNAs	Gen3G					3D				
	% Women with Detected miRNA	Normalized miRNA Levels Mean ± SD	L2FC	*p*-Value	q-Value	% Women with Detected miRNA	Normalized miRNA Levels Mean ± SD	L2FC	*p*-Value	q-Value
hsa-miR-516b-5p ^a^	99.31	101.64 ± 97.46	−0.634	1.16 × 10^−5^	0.005	100.00	280.99 ± 210.94	−0.585	0.0019	0.02
hsa-miR-1323 ^a^	99.77	146.42 ± 161.03	−0.652	1.18 × 10^−5^	0.005	100.00	574.92 ± 449.09	−0.631	0.0008	0.01
hsa-miR-512-3p ^a^	100.00	287.03 ± 379.56	−0.627	2.18 × 10^−5^	0.005	100.00	604.39 ± 470.70	−0.679	0.0003	0.01
hsa-miR-518e-5p|hsa-miR-519a-5p|hsa-miR-519b-5p|hsa-miR-519c-5p|hsa-miR-522-5p|hsa-miR-523-5p ^a^	98.62	44.04 ± 46.18	−0.602	2.63 × 10^−5^	0.005	98.11	87.19 ± 101.65	−0.788	0.0003	0.01
hsa-miR-520a-3p ^a^	99.31	86.87 ± 103.00	−0.622	3.49 × 10^−5^	0.005	99.06	172.45 ± 158.05	−0.865	0.00002	0.003
hsa-miR-519c-3p ^a^	89.68	11.08 ± 12.85	−0.700	3.57 × 10^−5^	0.005	92.45	31.11 ± 30.30	−0.817	0.0022	0.02
hsa-miR-517-5p ^a^	93.12	16.90 ± 19.37	−0.691	4.24 × 10^−5^	0.005	98.11	59.98 ± 51.93	−0.648	0.0021	0.02
hsa-miR-155-5p	100.00	916.98 ± 229.65	0.154	6.95 × 10^−5^	0.008	100.00	648.33 ± 291.30	0.205	0.0218	0.09
hsa-miR-516a-5p ^a^	96.56	31.30 ± 34.26	−0.609	9.71 × 10^−5^	0.01	99.06	121.46 ± 111.14	−0.626	0.0045	0.04
hsa-miR-515-3p ^a^	63.53	3.13 ± 4.59	−0.821	0.00022	0.02	82.08	13.07 ± 17.09	−0.855	0.0063	0.05
hsa-miR-515-5p ^a^	89.68	10.50 ± 12.77	−0.617	0.00024	0.02	90.57	21.35 ± 19.31	−0.623	0.0105	0.06
hsa-miR-1283 ^a^	98.39	57.28 ± 59.87	−0.544	0.00029	0.02	100.00	179.55 ± 178.70	−0.811	0.0001	0.009
hsa-miR-518e-3p ^a^	88.76	7.98 ± 9.25	−0.614	0.00050	0.03	94.34	25.60 ± 27.94	−0.883	0.0003	0.01
hsa-miR-526b-5p ^a^	94.72	17.11 ± 17.00	−0.540	0.00072	0.04	96.23	42.48 ± 45.67	−0.712	0.0017	0.02
hsa-miR-518a-5p|hsa-miR-527 ^a^	80.73	5.69 ± 7.14	−0.627	0.00074	0.04	83.02	13.29 ± 15.58	−1.197	0.00002	0.003
hsa-miR-525-5p ^a^	88.99	9.10 ± 10.59	−0.580	0.00097	0.05	96.23	67.12 ± 70.95	−0.838	0.0002	0.009
hsa-miR-524-5p ^a^	86.24	8.68 ± 9.93	−0.582	0.00139	0.06	96.23	45.02 ± 48.01	−0.893	0.00003	0.003
hsa-miR-145-3p	99.77	29.46 ± 18.44	−0.274	0.00262	0.0991	98.11	86.92 ± 60.59	−0.390	0.0136	0.07

Models adjusted for gestational age at blood collection time, as well as sequencing lane and run. ^a^ miRNAs from the C19MC cluster. Abbreviations: % women: percentage of women with at least one DESeq2-normalized read count; mean ± SD: mean and standard deviation of DESeq2-normalized read counts; L2FC: fold change per mmol/L in log2; *p*-value: unadjusted *p*-value; q-value: FDR-adjusted *p*-value.

**Table 3:** miRNAs associated with fasting glucose after correction for maternal age and BMI.

miRNAs	Gen3G				3D			
	% Women with Detected miRNA	Normalized miRNA Levels Mean ± SD	L2FC	*p*-Value	% Women with Detected miRNA	Normalized miRNA Levels Mean ± SD	L2FC	*p*-Value
**hsa-miR-516b-5p ^a^**	**99.31**	**101.64± 97.46**	**−0.361**	**0.02**	**100.00**	**280.99 ± 210.94**	**−0.432**	**0.03**
**hsa-miR-1323 ^a^**	**99.77**	**146.42 ± 161.03**	**−0.393**	**0.01**	**100.00**	**574.92 ± 449.09**	**−0.435**	**0.03**
**hsa-miR-512-3p ^a^**	**100.00**	**287.03 ± 379.56**	**−0.378**	**0.01**	**100.00**	**604.39 ± 470.70**	**−0.527**	**0.01**
**hsa-miR-518e-5p|hsa-miR-519a-5p|hsa-miR-519b-5p|hsa-miR-519c-5p|hsa-miR-522-5p|hsa-miR-523-5p ^a^**	**98.62**	**44.04 ± 46.18**	**−0.348**	**0.02**	**98.11**	**87.19 ± 101.65**	**−0.692**	**0.004**
**hsa-miR-520a-3p ^a^**	**99.31**	**86.87 ± 103.00**	**−0.371**	**0.02**	**99.06**	**172.45 ± 158.05**	**−0.795**	**0.0005**
**hsa-miR-519c-3p ^a^**	**89.68**	**11.08 ± 12.85**	**−0.491**	**0.006**	**92.45**	**31.11 ± 30.30**	**−0.697**	**0.02**
**hsa-miR-517-5p ^a^**	**93.12**	**16.90± 19.37**	**−0.466**	**0.009**	**98.11**	**59.98 ± 51.93**	**−0.604**	**0.01**
hsa-miR-155-5p	100.00	916.98± 229.65	0.113	0.006	100.00	648.33 ± 291.30	0.179	0.07
**hsa-miR-516a-5p ^a^**	**96.56**	**31.30 ± 34.26**	**−0.336**	**0.04**	**99.06**	**121.46 ± 111.14**	**−0.478**	**0.05**
**hsa-miR-515-3p ^a^**	**63.53**	**3.13 ± 4.59**	**−0.522**	**0.03**	**82.08**	**13.07 ± 17.09**	**−0.745**	**0.03**
hsa-miR-515-5p ^a^	89.68	10.50 ± 12.77	−0.419	0.02	90.57	21.35 ± 19.31	−0.528	0.05
hsa-miR-1283 ^a^	98.39	57.28 ± 59.87	−0.282	0.07	100.00	179.55 ± 178.70	−0.702	0.003
hsa-miR-518e-3p ^a^	88.76	7.98 ± 9.25	−0.296	0.1	94.34	25.60 ± 27.94	−0.754	0.005
hsa-miR-526b-5p ^a^	94.72	17.11 ± 17.00	−0.293	0.08	96.23	42.48 ± 45.67	−0.689	0.007
**hsa-miR-518a-5p|hsa-miR-527 ^a^**	**80.73**	**5.69 ± 7.14**	**−0.444**	**0.02**	**83.02**	**13.29 ± 15.58**	**−1.175**	**0.0002**
hsa-miR-525-5p ^a^	88.99	9.10 ± 10.59	−0.301	0.1	96.23	67.12 ± 70.95	−0.761	0.002
hsa-miR-524-5p ^a^	86.24	8.68 ± 9.93	−0.269	0.1	96.23	45.02 ± 48.01	−0.929	0.0001
hsa-miR-145-3p	99.77	29.46 ± 18.44	−0.180	0.06	98.11	86.92 ± 60.59	−0.369	0.03

Models adjusted for sequencing lane and run, as well as gestational age, maternal age, and BMI at blood collection. ^a^ miRNAs from the C19MC cluster. Abbreviations: % women: percentage of women with at least one DESeq2-normalized read count; mean ± SD: mean and standard deviation of DESeq2-normalized read counts; L2FC: fold change per mmol/L in log2; p-value: unadjusted *p*-value; q-value: FDR-adjusted *p*-value.

## Data Availability

Data are available on GEO (GSE216275 and GSE216997).

## Supplementary Materials

The following supporting information can be downloaded at: https://www.mdpi.com/article/10.3390/biomedicines12061285/s1, Table S1: Supplementary Table S1: Complete list of fasting glucose-associated miRNAs in Gen3G; Table S2: Supplementary Table S2: Sensibility analysis without participants with higher fasting glycemia; Table S3: Supplementary Table S3: miRNA associated with 1 h post-OGTT glycemia; Table S4: Supplementary Table S4: miRNAs associated with 2 h post-OGTT glycemia; Table S5: Supplementary Table S5: miRNAs associated with OGTT glycemia AUC values.
