# Supplementary material for: First-Trimester Plasmatic microRNAs Are Associated with Fasting Glucose Levels in Late Second Trimester of Pregnancy
Source: Biomedicines. 2024 Jun 10;12(6):1285. doi: 10.3390/biomedicines12061285 (PMC11201443; doi:10.3390/biomedicines12061285)
Supplement: Supplementary file 1 [file biomedicines-12-01285-s001.zip › Supplementary Table S1.pdf]

Supplementary Table S1: Complete list of fasting glucose associated miRNAs in Gen3G.

miRNA	% women with detected miRNA	Normalized miRNA levels Mean ± SD	L2FC	p-value	q-value
hsa-miR-1323[a]	99.77	146.42 ± 161.03	-0.652	1.18E-05	0.005
hsa-miR-516b-5p[a]	99.31	101.64 ± 97.46	-0.634	1.16E-05	0.005
hsa-miR-512-3p[a]	100.00	287.03 ± 379.56	-0.627	2.18E-05	0.005
hsa-miR-518e-5p|hsa-miR-519a-5p|hsa-miR-519b-5p|hsa-miR-519c-5p|hsa-miR-522-5p|hsa-miR-523-5p[a]	98.62	44.04 ± 46.18	-0.602	2.63E-05	0.005
hsa-miR-520a-3p[a]	99.31	86.87 ± 103.00	-0.622	3.49E-05	0.005
hsa-miR-519c-3p[a]	89.68	11.08 ± 12.85	-0.700	3.57E-05	0.005
hsa-miR-517-5p[a]	93.12	16.90 ± 19.37	-0.691	4.24E-05	0.005
hsa-miR-155-5p	100.00	916.98 ± 229.65	0.154	6.95E-05	0.008
hsa-miR-516a-5p[a]	96.56	31.30 ± 34.26	-0.609	9.71E-05	0.01
hsa-miR-517a-3p|hsa-miR-517b-3p[a]	95.41	20.18 ± 24.91	-0.606	0.0002	0.02
hsa-miR-515-3p[a]	63.53	3.13 ± 4.59	-0.821	0.0002	0.02
hsa-miR-515-5p[a]	89.68	10.50 ± 12.77	-0.617	0.0002	0.02
hsa-miR-1283[a]	98.39	57.28 ± 59.87	-0.544	0.0003	0.02
hsa-miR-517c-3p[a]	63.53	3.29 ± 4.47	-0.817	0.0003	0.02
hsa-miR-3191-3p	80.73	3.28 ± 2.82	0.462	0.0004	0.02
hsa-miR-518e-3p[a]	88.76	7.98 ± 9.25	-0.614	0.0005	0.03
hsa-miR-526b-5p[a]	94.72	17.11 ± 17.00	-0.540	0.0007	0.04
hsa-miR-518a-5p|hsa-miR-527[a]	80.73	5.69 ± 7.14	-0.627	0.0007	0.04
hsa-miR-525-5p[a]	88.99	9.10 ± 10.59	-0.580	0.0010	0.04
hsa-miR-524-5p[a]	86.24	8.68 ± 9.93	-0.582	0.0014	0.06
hsa-miR-519d-5p[a]	80.28	6.14 ± 7.10	-0.613	0.0017	0.07
hsa-miR-143-3p	100.00	31282.88 ± 14358.43	-0.229	0.0019	0.08
hsa-miR-524-3p[a]	86.24	8.68 ± 9.93	-0.697	0.0020	0.08
hsa-miR-145-3p	99.77	29.46 ± 18.44	-0.274	0.0026	0.099

Model adjusted for gestational age at blood collection time as well as sequencing lane and run. [a] miRNAs from the C19MC. Abbreviation: % women: percentage of women with at least one DESeq2 normalised read count; Mean ± SD: mean and standard deviation of DESeq2 normalised reads counts; L2FC: fold change in log_2; p-value: nominal p-value; q-value: FDR adjusted p-value.
