# Supplementary material for: First-Trimester Plasmatic microRNAs Are Associated with Fasting Glucose Levels in Late Second Trimester of Pregnancy
Source: Biomedicines. 2024 Jun 10;12(6):1285. doi: 10.3390/biomedicines12061285 (PMC11201443; doi:10.3390/biomedicines12061285)
Supplement: Supplementary file 1 [file biomedicines-12-01285-s001.zip › Supplementary Table S2.pdf]

Supplementary Table S2: Sensibility analysis without the participant with higher fasting glycemia

miRNA	Model 1			Model 2	
	L2FC	p-value	q-value	L2FC	p-value
hsa-miR-1323[a]	-0.606	7.94E-05	0.02	-0.321	0.05
hsa-miR-516b-5p[a]	-0.664	2.92E-05	0.02	-0.388	0.02
hsa-miR-512-3p[a]	-0.565	0.0003	0.02	-0.310	0.06
hsa-miR-518e-5p|hsa-miR-519a-5p|hsa-miR-519b-5p|hsa-miR-519c-5p|hsa-miR-522-5p|hsa-miR-523-5p[a]	-0.557	0.0002	0.02	-0.294	0.07
hsa-miR-520a-3p[a]	-0.570	0.0003	0.02	-0.312	0.06
hsa-miR-519c-3p[a]	-0.677	9.45E-05	0.02	-0.463	0.01
hsa-miR-517-5p[a]	-0.651	0.0002	0.02	-0.421	0.02
hsa-miR-155-5p	0.144	0.0005	0.03	0.099	0.02
hsa-miR-516a-5p[a]	-0.564	0.0005	0.03	-0.285	0.096
hsa-miR-515-3p[a]	-0.842	0.0003	0.02	-0.539	0.03
hsa-miR-515-5p[a]	-0.648	0.0002	0.02	-0.446	0.01
hsa-miR-1283[a]	-0.481	0.0024	0.098	-0.213	0.2
hsa-miR-518e-3p[a]	-0.584	0.0013	0.06	-0.259	0.2
hsa-miR-526b-5p[a]	-0.495	0.0029	0.1	-0.243	0.2
hsa-miR-518a-5p|hsa-miR-527[a]	-0.614	0.0013	0.06	-0.427	0.04
hsa-miR-525-5p[a]	-0.551	0.0023	0.098	-0.267	0.2
hsa-miR-524-5p[a]	-0.553	0.0031	0.1	-0.231	0.2
hsa-miR-145-3p	-0.239	0.0125	0.3	-0.139	0.2

Model 1: adjusted for gestational age at blood collection time as well as sequencing lane and run. Model 2: adjusted for gestational age at blood collection time as sequencing lane and run as well as maternal age and BMI. [a]miRNAs from the C19MC cluster. Abbreviations: L2FC: fold change per mmol/L in log2; p-value: unadjusted p-value; q-value: FDR adjusted p-value:
