# Supplementary material for: First-Trimester Plasmatic microRNAs Are Associated with Fasting Glucose Levels in Late Second Trimester of Pregnancy
Source: Biomedicines. 2024 Jun 10;12(6):1285. doi: 10.3390/biomedicines12061285 (PMC11201443; doi:10.3390/biomedicines12061285)
Supplement: Supplementary file 1 [file biomedicines-12-01285-s001.zip › Supplementary Table S3.pdf]

## Supplementary Table S3: miRNA associated with 1 hour post-OGTT glycemia.

| miRNA | Gen3G | | | | | 3D | | | | |
|---|---|---|---|---|---|---|---|---|---|---|
| | % women with detected miRNA | Normalized miRNA levels Mean ± SD | L2FC | p-value | q-value | % women with detected miRNA | Normalized miRNA levels Mean ± SD | L2FC | p-value | q-value |
| **Model adjusted for gestational age at first trimester, as well as sequencing lane and run** | | | | | | | | | | |
| hsa-miR-143-3p | 100.00 | 31282.88 ± 14358.43 | -0.085 | 6.73E-07 | 0.001 | 100.00 | 49304.7± 99720.91 | 0.0 33 | 0.6114 | 1.00 |
| **Model adjusted for gestational age at first trimester, sequencing lane and run, as well as maternal age and BMI at first trimester** | | | | | | | | | | |
| hsa-miR-143-3p | 100.00 | 31282.88 ± 14358.43 | -0.075 | 2.41E-05 | 0.05 | 100.00 | 49304.7± 99720.91 | 0.0 48 | 0.460 | 1.00 |

Abbreviations: % women: percentage of women with at least one DESeq2 normalised read count; Mean ± SD: mean and standard deviation of DESeq2 normalised reads counts; L2FC: fold change in $log_2$; p-value: nominal p-value; q-value: FDR adjusted p-value.
