# Supplementary material for: First-Trimester Plasmatic microRNAs Are Associated with Fasting Glucose Levels in Late Second Trimester of Pregnancy
Source: Biomedicines. 2024 Jun 10;12(6):1285. doi: 10.3390/biomedicines12061285 (PMC11201443; doi:10.3390/biomedicines12061285)
Supplement: Supplementary file 1 [file biomedicines-12-01285-s001.zip › Supplementary Table S4.pdf]

Supplementary Table S4: miRNAs associated with 2 hours post-OGTT glycemia.

miRNAs	Gen3G					3D				
	% women with detected miRNA	Normalized miRNA levels Mean ± SD	L2FC	p-value	q-value	% women with detected miRNA	Normalized miRNA levels Mean ± SD	L2FC	p-value	q-value
**Model adjusted for gestational age at first trimester, as well as sequencing lane and run**										
hsa-miR-143-3p	100.00	31282.88 ± 14358.43	-0.090	2.13E-05	0.01	100.00	49304.7± 99720.91	-0.153	0.0287	1.00
hsa-miR-484	100.00	2659.32 ± 850.39	0.058	2.35E-05	0.01	100.00	6694.33 ± 3786.75	0.088	0.0240	1.00
hsa-miR-183-5p	100.00	330.59 ± 304.27	0.148	0.0001	0.03	100.00	364.76 ± 345.36	0.083	0.2890	1.00
hsa-miR-200a-3p	99.54	29.35 ± 44.66	-0.144	0.0002	0.03	98.11	44.77 ± 39.25	0.038	0.6302	1.00
hsa-miR-141-3p	100.00	147.19 ± 240.04	-0.141	0.0002	0.03	100.00	452.03 ± 357.75	-0.024	0.7289	1.00
hsa-miR-182-5p	100.00	22298.89 ± 17704.17	0.125	0.0002	0.03	100.00	11284.07 ± 10384.04	-0.038	0.5452	1.00
hsa-miR-1304-3p	42.89	17.99 ± 99.35	-0.425	0.0006	0.08	8.49	16.51 ± 98.16	0.525	0.3656	1.00
hsa-miR-486-5p	100.00	2441401.98 ± 2184247.77	0.125	0.0006	0.08	100.00	4494867.69 ± 8106991.94	-0.001	0.9976	1.00
hsa-miR-338-3p	100.00	108.46 ± 36.14	-0.051	0.0006	0.08	100.00	217.91 ± 87.15	-0.002	0.9626	1.00
hsa-miR-483-5p	99.54	36.57 ± 32.85	-0.127	0.0006	0.08	100.00	88.89 ± 57.73	0.008	0.8892	1.00
hsa-miR-421	100.00	992.8 ± 228.23	0.036	0.0007	0.08	100.00	1453.97 ± 252.67	-0.008	0.6398	1.00
hsa-miR-4477b	43.12	1.24 ± 1.63	0.210	0.0009	0.09	21.7	1.61 ± 7.7	-0.620	0.0357	1.00
hsa-miR-18a-3p	100.00	175.77 ± 122.72	0.089	0.001	0.09	100.00	182.01 ± 119.75	0.026	0.6186	1.00
hsa-miR-7706	100.00	158.26 ± 98.57	0.080	0.001	0.098	100.00	203.95 ± 135.43	-0.007	0.8828	1.00
**Model adjusted for gestational age at first trimester, sequencing lane and run, as well as maternal age and BMI at first trimester**										
hsa-miR-143-3p	100.00	31282.88 ± 14358.43	-0.079	0.0002	0.02	100.00	49304.7± 99720.91	-0.148	0.0356	1.00
hsa-miR-484	100.00	2659.32 ± 850.39	0.058	2.71E-05	0.01	100.00	6694.33 ± 3786.75	0.091	0.0214	1.00
hsa-miR-183-5p	100.00	330.59 ± 304.27	0.155	8.41E-05	0.01	100.00	364.76 ± 345.36	0.079	0.3207	1.00
hsa-miR-200a-3p	99.54	29.35 ± 44.66	-0.124	0.0013	0.05	98.11	44.77 ± 39.25	0.045	0.5481	1.00
hsa-miR-141-3p	100.00	147.19 ± 240.04	-0.113	0.0028	0.07	100.00	452.03 ± 357.75	-0.017	0.8028	1.00
hsa-miR-182-5p	100.00	22298.89 ± 17704.17	0.137	5.80E-05	0.01	100.00	11284.07 ± 10384.04	-0.038	0.5521	1.00
hsa-miR-1304-3p	42.89	17.99 ± 99.35	-0.112	0.7148	0.96	8.49	16.51 ± 98.16	0.064	0.9503	1.00
hsa-miR-486-5p	100.00	2441401.98 ± 2184247.77	0.137	0.0002	0.02	100.00	4494867.69 ± 8106991.94	-0.002	0.9896	1.00

hsa-miR-338-3p	100.00	108.46 ± 36.14	-0.049	0.0013	0.05	100.00	217.91 ± 87.15	0.006	0.8829	1.00
hsa-miR-483-5p	99.54	36.57 ± 32.85	-0.107	0.0042	0.098	100.00	88.89 ± 57.73	0.037	0.5347	1.00
hsa-miR-421	100.00	992.8 ± 228.23	0.036	0.0009	0.04	100.00	1453.97 ± 252.67	-0.012	0.4811	1.00
hsa-miR-4477b	43.12	1.24 ± 1.63	0.214	0.0010	NA	21.7	1.61 ± 7.7	-0.722	0.0149	1.00
hsa-miR-18a-3p	100.00	175.77 ± 122.72	0.098	0.0004	0.02	100.00	182.01 ± 119.75	0.030	0.5612	1.00
hsa-miR-7706	100.00	158.26 ± 98.57	0.091	0.0003	0.02	100.00	203.95 ± 135.43	-0.010	0.8314	1.00

Abbreviations: % women: percentage of women with at least one DESeq2 normalised read count; Mean ± SD: mean and standard deviation of DESeq2 normalised reads counts; L2FC: fold change in $\log_2$; p-value: nominal p-value; q-value: FDR adjusted p-value.
