# Supplementary material for: First-Trimester Plasmatic microRNAs Are Associated with Fasting Glucose Levels in Late Second Trimester of Pregnancy
Source: Biomedicines. 2024 Jun 10;12(6):1285. doi: 10.3390/biomedicines12061285 (PMC11201443; doi:10.3390/biomedicines12061285)
Supplement: Supplementary file 1 [file biomedicines-12-01285-s001.zip › Supplementary Table S5.pdf]

Supplementary Table S5: miRNAs associated with OGTT glycemia AUC values.

miRNAs	Gen3G					3D				
	% women with detected miRNA	Normalized miRNA levels Mean ± SD	L2FC	p-value	q-value	% women with detected miRNA	Normalized miRNA levels Mean ± SD	L2FC	p-value	q-value
**Model adjusted for gestational age at first trimester, as well as sequencing lane and run**										
hsa-miR-143-3p	100.00	31282.88 ± 14358.43	-0.066	1.36E-07	0.0003	100.00	49304.7± 99720.91	-0.003	0.9	1.00
hsa-miR-484	100.00	2659.32 ± 850.39	0.032	7.89E-05	0.08	100.00	6694.33± 3786.75	0.012	0.6	1.00
**Model adjusted for gestational age at first trimester, sequencing lane and run, as well as maternal age and BMI at first trimester**										
hsa-miR-143-3p	100.00	31282.88 ± 14358.43	-0.059	7.68E-06	0.02	100.00	49304.7± 99720.91	0.006	0.9	1.00
hsa-miR-484	100.00	2659.32 ± 850.39	0.034	6.02E-05	0.06	100.00	6694.33± 3786.75	0.014	0.6	1.00

Abbreviations: % women: percentage of women with at least one DESeq2 normalised read count; Mean ± SD: mean and standard deviation of DESeq2 normalised reads counts; L2FC: fold change in log_2; p-value: nominal p-value; q-value: FDR adjusted p-value.
